# Integrated Microbiomic and Metabolomic Dynamics of Fermented Corn and Soybean By-Product Mixed Substrate

**DOI:** 10.3389/fnut.2022.831243

**Published:** 2022-02-28

**Authors:** Cheng Wang, Siyu Wei, Mingliang Jin, Bojing Liu, Min Yue, Yizhen Wang

**Affiliations:** ^1^National Engineering Laboratory for Feed Safety and Pollution Prevention and Controlling, Hangzhou, China; ^2^Key Laboratory of Molecular Animal Nutrition, Ministry of Education, Hangzhou, China; ^3^Key Laboratory of Animal Nutrition and Feed Science (Eastern of China), Ministry of Agriculture and Rural Affairs, Hangzhou, China; ^4^Key Laboratory of Animal Feed and Nutrition of Zhejiang Province, Hangzhou, China; ^5^Institute of Feed Science, Zhejiang University, Hangzhou, China; ^6^Institute of Preventive Veterinary Sciences and Department of Veterinary Medicine, Zhejiang University College of Animal Sciences, Hangzhou, China

**Keywords:** fermentation, metabolomic analyses, dynamics, microbiomic data, plant-based food by-product

## Abstract

Microbes and their metabolites produced in fermented food have been considered as critical contributors to the quality of the final products, but the comprehensive understanding of the microbiomic and metabolomic dynamics in plant-based food during solid-state fermentation remains unclear. Here, the probiotics of *Bacillus subtilis* and *Enterococcus faecalis* were inoculated into corn and defatted soybean to achieve the two-stage solid-state fermentation. A 16S sequencing and liquid chromatography–tandem mass spectrometry were applied to investigate the dynamics of microbiota, metabolites, and their integrated correlations during fermentation. The results showed that the predominant bacteria changed from *Streptophyta and Rickettsiales* at 0 h to *Bacillus* and *Pseudomonas* in aerobic stage and then to *Bacillus, Enterococcus*, and *Pseudomonas* in anaerobic stage. In total, 229 notably different metabolites were identified at different fermentation times, and protein degradation, amino acid synthesis, and carbohydrate metabolism were the main metabolic pathways during the fermentation. Notably, phenylalanine metabolism was the most important metabolic pathway in the fermentation process. Further analysis of the correlations among the microbiota, metabolites, and physicochemical characteristics indicated that *Bacillus* spp. was significantly correlated with amino acids and carbohydrate metabolism in aerobic stage, and *Enterococcus* spp. was remarkably associated with amino acids metabolism and lactic acid production in the anaerobic stage. The present study provides new insights into the dynamic changes in the metabolism underlying the metabolic and microbial profiles at different fermentation stages, and are expected to be useful for future studies on the quality of fermented plant-based food.

## Introduction

Corn is one of the main grains produced worldwide, providing 30% of food calories for more than 4.5 billion people worldwide, and is considered to be the main staple food in most countries (1, 2). Whereas, compared with other cereal crops, the nutritional value of corn is low, lacking essential amino acids and lysine (3). Soy-derived foods have been consumed for centuries, especially in Asian diets. But soy products contain isoflavones, antigen proteins, and fiber substances that are not easy to be absorbed, which affect their bioavailability (4, 5). Therefore, many processing technologies like physical and chemical methods have been applied to corn and soybean-based foods to enhance the nutritional value of the final products. However, these processing tools are un-environmental friendly and lose nutrients (6).

Fermentation is one of the traditional biotechnologies in the food field because it makes a solid foundation for the development of safe food with better nutrition and functions. It is a natural way to preserve food, increase nutritional value and digestibility, and reduce anti-nutritional factors. Fermenting microbes like *Bacillus* and *Aspergillus* can degrade anti-nutritional factors and improve food quality (7, 8). Lactic acid bacteria can produce organic acids, improve food flavor, and lengthen storage time (9, 10). In the past few years, many studies have been conducted on fermentation to obtain fermented corn-based products that are beneficial to human health (11–13). In addition, fermentation has been used to improve the bioavailability of protein, vitamins, minerals, and isoflavones in soybeans, as well as to change their flavor, improve stability, and even create novel foods (14–17). Natto, fermented bean curd, Miso, and soy sauce have a long history in the Asian diet. It has been reported that these traditional fermented soybean-based foods can improve bone health, reduce the risk of cancer, and prevent the progression of diabetes (18).

Fermentation is conducted through the action of various microorganisms under open or semi-open conditions, including starters and local microorganisms, and plays important roles in anti-nutritional factor degradation, nutrition improvement, and flavor production (19, 20). At present, high-throughput sequencing provides a reliable method for the comprehensive description of microbial community dynamics and expands our understanding of the microbial community structure of fermented corn and soybean products. The latest research showed that *Lactococcus, Streptococcus, Enterobacter, Acinetobacter*, and fungi such as *Trichosporon* and *Aspergillus* are common fermenting microbes in Sufu flora (21). In addition, microbial abundance is mainly related to nutritional characteristics. Besides, metabolomics methods such as gas chromatography (GC), mass spectrometry (MS), and high-performance liquid chromatography (HPLC) have been applied to determine the metabolite profiles of fermented foods (22, 23). However, the comprehensive dynamic changes of microbes and metabolites during plant-based food fermentation still lack understanding, especially in corn and defatted soybean. Here, corn and defatted soybean were carried out with a two-stage solid-state fermentation inoculating with *Bacillus subtilis* and *Enterococcus faecalis*. A 16S rRNA sequencing and liquid chromatography–mass spectrometry (LC/MS) were used to explore the microbial and metabolic dynamics during the fermentation. The evidence will expand our knowledge of improving the quality of fermented plant-based food from an integrated microbiomic and metabolomic perspective.

## Materials and Methods

### Experimental Design and Sampling

*Bacillus subtilis* (NCBI Accession No. MH885533, CGMCC No:12825) and *Enterococcus faecalis* (NCBI Accession No. MN038173, CGMCC 1.15424) were cultured, respectively, in Luria broth and de Man, Rogosa, and Sharpe liquid medium at 37°C, as described in detail by Wang et al. (24). About 200 g of the substrates contained 40% of defatted soybean, 40% of corn, and 20% of yellow wine lees were transferred into a 500 mL Erlenmeyer flask. Sterilized water was added to achieve a 40% moisture concentration. *B. subtilis* (1 × 10^7^ CFU/g) was inoculated in the mixed substrate at 37°C for 24 h in aerobic fermentation, and then *E. faecalis* (2 × 10^7^ CFU/g) was inoculated in the fermented substrate with an anaerobic fermentation at 37°C until 48 h. Samples were collected at 0, 12, 24, and 48 h. The nutrition determination of the fermented matrix is shown in Supplementary Table S1.

### The Measurement of Nutritional Content

Moist samples (~100 g) at 0, 12, 24, and 48 h were collected to determine the numbers of microorganisms and microbial metabolites and for 16S rRNA gene sequencing, and the remaining samples were dried at 60°C for 24 h, cooled, ground, and subjected to physicochemical analysis. Dried samples were collected for further analysis of the crude protein (CP), neutral detergent fiber (NDF), acid detergent fiber (ADF), and amylose contents using AOAC International guidelines (25). The content of phytate phosphorus was analyzed according to the method described by Shi et al. (26). The pH of the fermented product was determined with a pH meter (Mettler Toledo, Switzerland) according to a previous study (27). Lactate was detected using a lactic acid assay kit (Nanjing Jiancheng Bioengineering Institute, Nanjing) following the manufacturer's instructions. The contents of antigenic protein were analyzed using a commercial kit (Jiangsu Meibiao Biological Technology Co., Jiangsu, China).

### Liquid Chromatography–Mass Spectrometry Analysis and Data Processing

A 50 mg of the sample was weighted to an EP tube, and 1,000 μL extract solution (methanol:acetonitrile:water = 2:2:1, with isotopically labeled internal standard mixture) was added. Then the samples were homogenized at 35 Hz for 4 min and sonicated for 5 min in ice water bath. The homogenization and sonication cycle was repeated 3 times. Then the samples were incubated for 1 h at −40°C and centrifuged at 12,000 rpm for 15 min at 4°C. The resulting supernatant was transferred to a fresh glass vial for analysis. The quality control (QC) sample was prepared by mixing an equal aliquot of the supernatants from all of the samples.

LC–MS/MS analyses were performed using a UHPLC system (Vanquish, Thermo Fisher Scientific) with a UPLC BEH Amide column (2.1 mm × 100 mm, 1.7 μm) coupled to Q Exactive HFX mass spectrometer (Orbitrap MS, Thermo). The mobile phase consisted of 25 mmol/L ammonium acetate and 25 ammonia hydroxide in water (pH = 9.75) (A) and acetonitrile (B). The auto-sampler temperature was 4°C, and the injection volume was 3 μL. The QE HFX mass spectrometer was used for its ability to acquire MS/MS spectra on information-dependent acquisition (IDA) mode in the control of the acquisition software (Xcalibur, Thermo). In this mode, the acquisition software continuously evaluates the full scan MS spectrum. The ESI source conditions were set as follows: sheath gas flow rate as 30 Arb, Aux gas flow rate as 25 Arb, capillary temperature 350°C, full MS resolution as 60,000, MS/MS resolution as 7,500, collision energy as 10/30/60 in NCE mode, spray Voltage as 3.6 kV (positive) or −3.2 kV (negative), respectively.

The raw data were converted to the mzXML format using ProteoWizard and processed with an in-house program, which was developed using R and based on XCMS, for peak detection, extraction, alignment, and integration. Then an in-house MS2 database (BiotreeDB) was applied in metabolite annotation. The cutoff for annotation was set at 0.3. The obtained data were conducted to principal component analysis (PCA), Partial Least Squares Discriminant Analysis (PLS-DA), and orthogonal partial least squares discriminant analysis (OPLS-DA). The goodness-of-fit parameter (R2) and the predictive ability parameter (Q2) were used to evaluate the quality of PLS-DA and OPLS-DA models. Variable importance projection (VIP) > 1.0 and *P* < 0.05 were identified as the differential metabolites. To further interpret the biological significance of metabolites, metabolic pathway analyses were performed by an online analysis platform in the MetaboAnalyst 5.0 (https://www.metaboanalyst.ca/). KEGG analysis has been conducted by enrichment analysis sections of MetaboAnalyst.

### Bioinformatics Analysis of Sequencing Data

Total DNA was extracted from 16 samples using the E.Z.N.A. soil DNA kit (Omega Bio-Tek, Norcross, GA, United States). A NanoDrop 2,000 UV–vis spectrophotometer (Thermo Scientific, Wilmington, DE, United States) and 1% agarose gel electrophoresis were used to analyze DNA content and quality.

The MiSeq platform (Personal Biotechnology Co., Ltd, Shanghai, China) was used to describe the bacterial community based on the gene segment from the the V3-V4 gene regions of the bacterial 16S rRNA gene primers 338F (5′-ACTCCTACGGGAGGCAGCAG-3′) and 806R (5′-GGACTACHVGGGTWTCTAAT-3′). PCR was conducted as follows: 3 min of denaturation at 95°C; 27 cycles of 30 s at 95°C, 30 s for annealing at 55°C, and 45 s for elongation at 72°C; and a final extension at 72°C for 10 min. The AxyPrep DNA gel extraction kit (Axygen Biosciences, Union City, CA, United States) and QuantiFluor-ST instrument (Promega, United States) were used to further extract, purify, and quantify the PCR products. Subsequently, raw Illumina FASTQ files were demultiplexed, quality filtered, and analyzed using Quantitative Insights into Microbial Ecology (QIIME v1.9.1). Raw fastq files were quality filtered by Trichromatic and merged by FLASH. OTUs were clustered with a 97% similarity cutoff using UPARSE (version 7.1). The taxonomy of each 16S rRNA gene sequence was analyzed using the RDP Classifier algorithm (http://rdp.cme.msu.edu/) against the Silvia 16S rRNA database using a confidence threshold of 70%. The assembled MiSeq sequences were submitted to the NCBI's Sequence Read Archive (SRA BioProject no. PRJNA552228) for open access. Estimates of diversity values for these samples using the Chao1, Shannon, and Simpson indexes for diversity estimation were calculated by rarefaction analysis. Nonmetric Multidimensional Scaling (NMDS) and cluster analysis with the ANOSIM method were conducted using the Web server tool METAGENassist based on unweighted UniFrac distances.

The main differentially abundant genera were selected by the LEfSe method (https://huttenhower.sph.harvard.edu/galaxy/). To predict metabolic genes during the process, PICRUSt (https://huttenhower.sph.harvard.edu/galaxy/) was applied to obtain a functional profile from the 16S rRNA data. Before metagenome prediction, the OTUs of 16S rRNA sequences were analyzed using PICRUSt. PICRUSt and KEGG were used to obtain functions for the genes that were predicted to be present in the samples and to assign the genes into metabolic pathways.

Spearman's rank correlation coefficient was calculated with R version 3.6.3 to evaluate the relationship among physicochemical characteristics, microbiota, and metabolites.

### Statistical Analysis

All the data were presented as means ± SEM (*N* = 4 for chemical and microbial analysis, *N* = 6 for metabolic analysis). The data were analyzed applying the SPSS software (version 26.0, SPSS Inc. Chicago, IL, United States). Statistical differences between different fermentation stages were determined by Student's *t*-tests and one-way ANOVA followed by Duncan's multiple-range test. The *P*-values of the metabolomics and microbiome data were corrected using Welch's test and the Benjamini–Hochberg false-discovery rate (FDR). *P*-values <0.05 indicate statistically significant differences. Bar plots were generated in GraphPad Prism 8 (San Diego, CA, United States).

## Results

### The Change of Physicochemical Parameters and Microbial Community

The nutrients are presented in Supplementary Table S1. The pH value showed a dramatic decrease from aerobic to anaerobic fermentation stage while the content of lactic acid increased approximately three-fold range at 48 h. The crude protein (CP) content significantly increased from 28.21 to 31.54%, and a sharp increase was observed in small peptides (SP). Notably, similar significantly downward trends were observed for the levels of amylose, NDF, phytate phosphorus, glycinin, and β-conglycinin during the whole process of fermentation.

**Figure 1:**
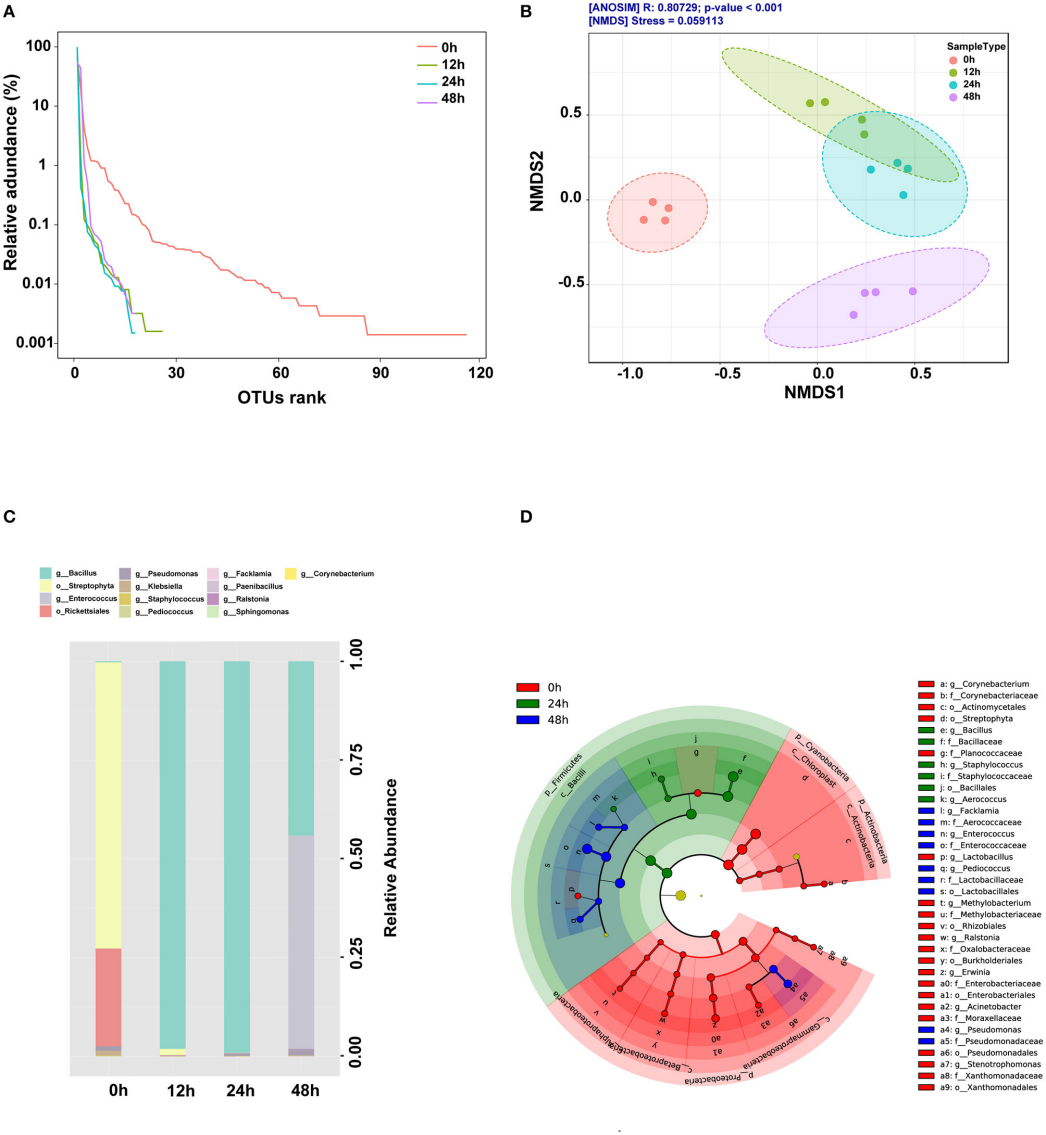
Changes of microbial community during fermentation. **(A)** OTU rank curve during fermentation. **(B)** β-diversity was performed using the ordination-based Nonmetric Multidimensional Scaling (NMDS) based on the Jaccard index distance method and analysis of group similarities (ANOSIM) at the species level. **(C)** Genus-level compositions of the bacterial community during fermentation. **(D)** Cladogram plot of significant genera based on LEfSe analysis (LDA scores >3.0).

The composition of the bacterial community during corn and defatted soybean fermentation is shown in Figure 1. α-Diversity is used to measure the diversity within a sample or an ecosystem. The two most commonly used alpha-diversity measurements are Richness (count) and Evenness (distribution). The rank-curve generated by OTU ranks and their relative abundance illustrated that α-diversity reduced during the initial 24 h after *B. subtilis* treatment, whereas increased at the anaerobic fermentation stage (Figure 1A). β-Diversity represents the explicit comparison of microbial communities (in-between) based on their composition and provides a measure of the distance or dissimilarity between each sample pair. The result showed that microbial β-diversity based on NMDS and Jaccard index distance method was distinct at different time points since the structures of microbial communities were separated into four clusters (Figure 1B). The results showed that the predominant bacteria changed from Streptophyta and Rickettsiales at 0 h to Bacillus and Pseudomonas in the aerobic stage and then to Bacillus, Enterococcus, and Pseudomonas in the anaerobic stage (Figure 1C). Significantly different bacteria from order to genus level among different fermentation time points were identified by the linear discriminant analysis effect size (LEfSe) (Figure 1D). The abundance of *Bacillus, Staphylococcus*, and *Aerococcus* increased significantly at 24 h. *Enterococcus, Pseudomonas, Pediococcus*, and *Facklamia* were the predominant genus at 48 h.

### Cluster Analysis of Metabolites at Different Fermentation Time Points

Unsupervised mode of the PCA can reveal the variation between and within groups, and reflect the tendency of distribution as well as possible discrete points. The first principal component (PC1 = 67.0%) and the second principal component (PC2 = 16.5%) directly showed the similarities and differences between groups (Supplementary Figure S1A). The result showed that fermentation samples at 0, 12, 24, and 48 h were separated, especially by the first principal component, and all samples fell within a 95% confidence ellipse. The accurate differences between samples could not be completely interpreted according to the visual distinctions generated by the PCA plot, because PCA is an unsupervised model and belongs to exploratory analysis. Therefore, supervised classification models of Partial Least Squares Discriminant Analysis (PLS-DA) and Orthogonal Partial Least Squares Discriminant Analysis (OPLS-DA) were implemented to confirm the transformation degree of metabolites with fermentation time. The results showed obvious separation between different fermentation groups indicating that it could be used to identify the metabolic differences in the fermentation process of the substrates (Supplementary Figures S1B,C). A further cluster analysis was to determine whether metabolites differences existed during the fermentation. The chart showed that all the samples were clustered into four groups, and each cluster with samples belonged to the same time points (Supplementary Figure S1D). These results suggested that good stability and reproducibility were obtained, and the change of metabolites during fermentation was time dependent.

### The Changes of Metabolic Composition at Different Levels

**Figure 2:**
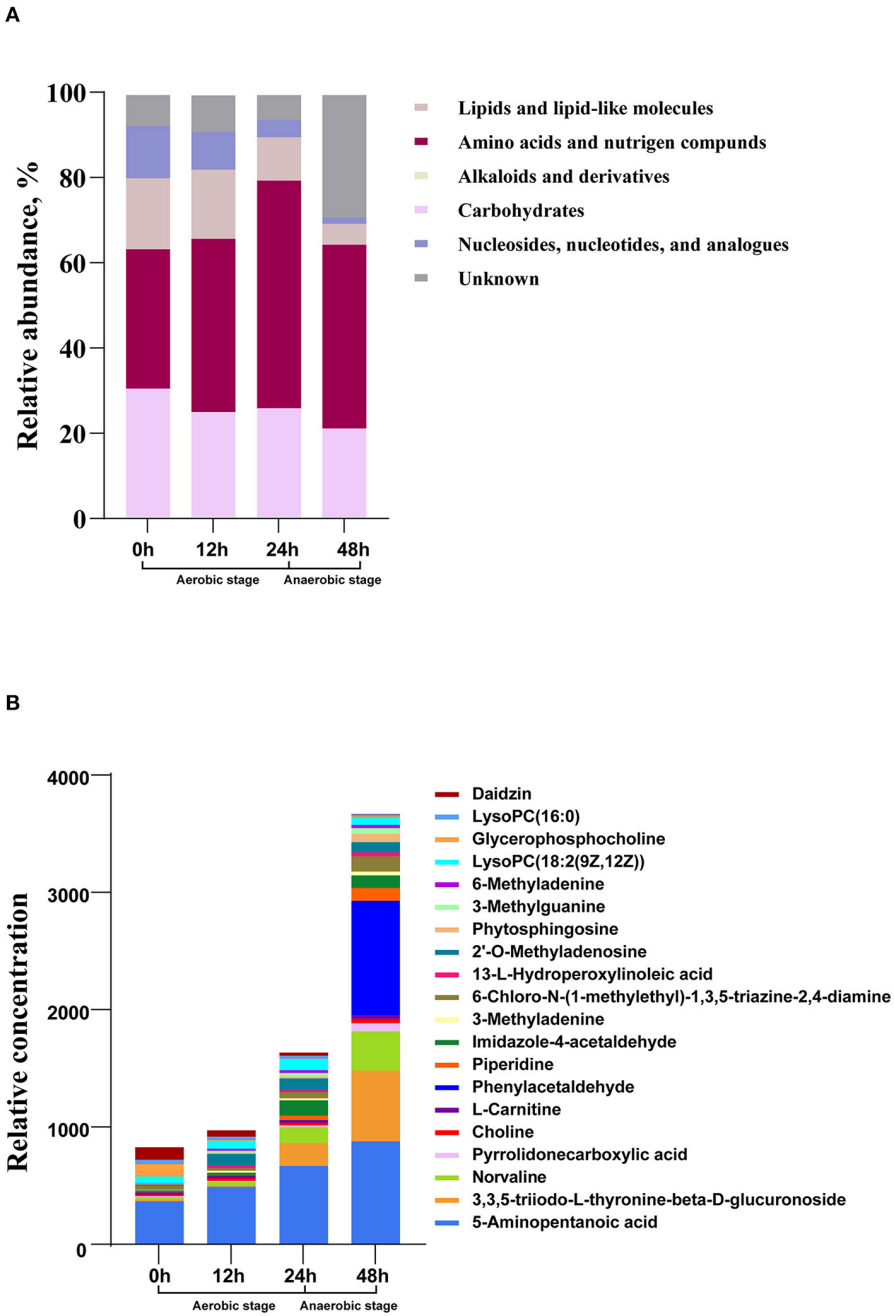
The changes of metabolic composition at different levels in the process of fermentation. **(A)** Relative compositions of the main metabolites. **(B)** Top 20 metabolites at different fermentation times.

Metabolome analysis revealed that nucleosides, nucleotides, and analogs; lipids and lipid-like molecules; carbohydrates; amino acids, peptides, and proteins were the main abundant metabolites during the whole fermentation stage (Figure 2A). The relative abundance of amino acids, peptides, and proteins in the process of fermentation constantly increased (*P* < 0.05), whereas the abundance of nucleosides, nucleotides, and analogs and lipids and lipid-like molecules maintained decreasing (*P* < 0.05). Carbohydrates decreased from 0 to 12 h and then remained in a relatively stable state. To find the specific change of the related metabolites, we explored the next level of top 20 metabolites (Figure 2B). At 0 h, daidzin, lysoPC(16:0), glycerophosphocholine, lysoPC[18:2(9Z,12Z)], and 5-aminopentanoic acid were the main metabolites. The top 5 metabolites at 12 h were daidzin, norvaline, lysoPC[18:2(9Z,12Z)], 2′-O-methyladenosine, and 5-aminopentanoic acid. Norvaline, 3,3,5-triiodo-L-thyronine-beta-D-glucuronoside, 5-aminopentanoic acid, imidazole-4-acetaldehyde, lysoPC[18:2(9Z,12Z)], and 2′-O-methyladenosine were the dominant substances at 24 h. Notably, the concentration of metabolites was sharply elevated in the anaerobic stage. Phenylacetaldehyde dramatically raised in the anaerobic stage and became the predominant metabolites. 3,3,5-Triiodo-L-thyronine-beta-D-glucuronoside and norvaline in samples at 48 h were higher relative to that in 24 h. During the whole fermentation stage, daidzin continued to reduce while 5-aminopentanoic acid persistently increased.

### Different Metabolites in the Process of Fermentation

**Figure 3:**
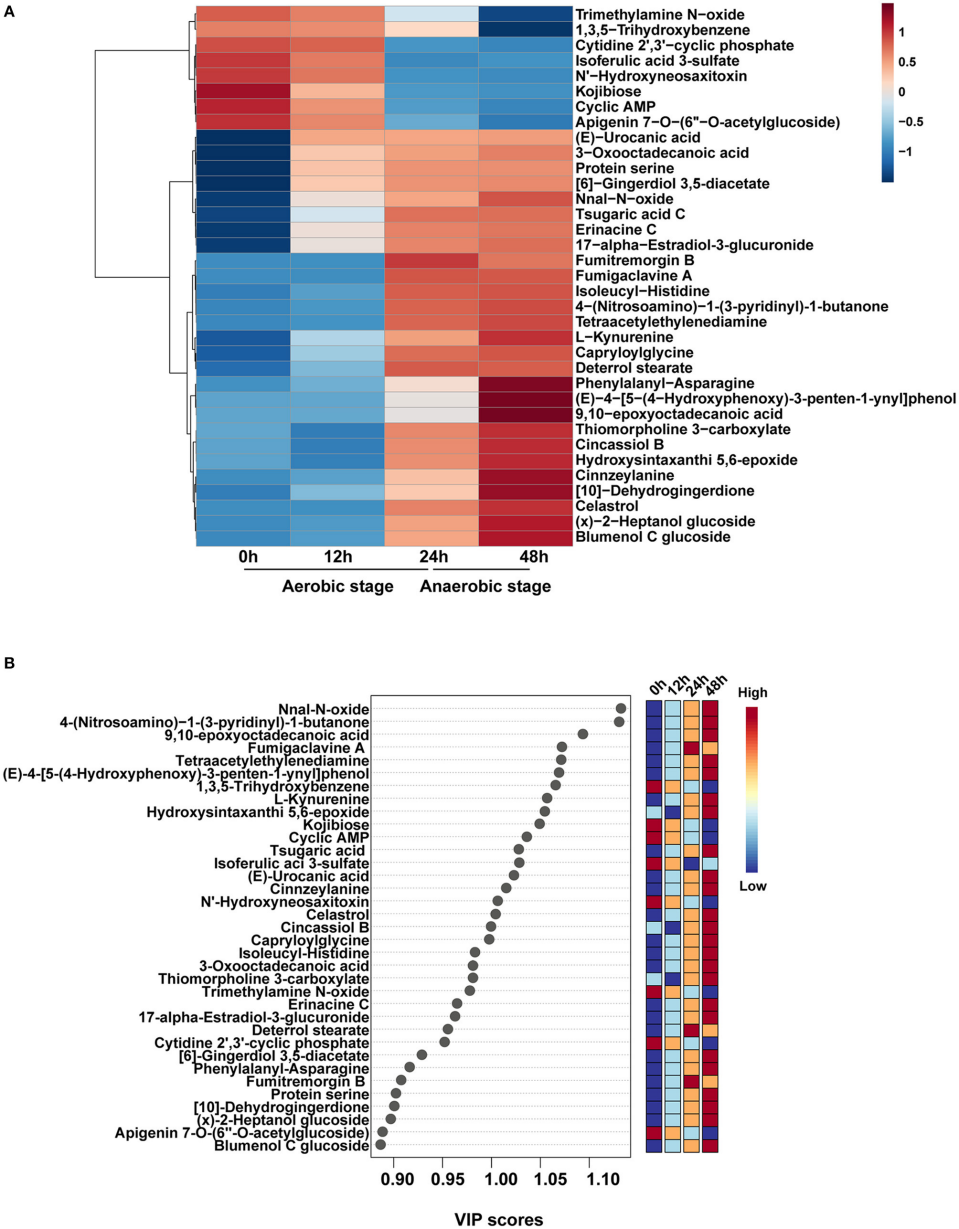
Significantly different metabolites during fermentation. **(A)** Top 35 significantly different metabolites. **(B)** A scatter plot of the top 35 distinct metabolites was identified by applying variable importance projection (VIP).

The main metabolites based on the variable importance for the projection (VIP) were selected and sorted to better understand the changes of the primary different metabolites. Totally 229 metabolites were identified after annotation, and these metabolites have individual metabolic features at different time points after fermentation. Top 35 significantly different metabolites during fermentation were selected (Figure 3A), and the VIP values of these 35 metabolites based on the PLS-DA model were shown (Figure 3B). The main significantly different metabolites enriched in 0 h were trimethylamine N–oxide, 1,3,5–trihydroxybenzene, cytidine 2′,3′–cyclic phosphate, isoferulic acid 3-sulfate, N'–hydroxyneosaxitoxin, kojibiose, cyclic AMP, and apigenin 7–O–(6″–O-acetylglucoside). These eight metabolites slightly reduced from 0 to 12 h while remarkably decreased in 24 and 48 h (*P* < 0.05). Fumigaclavine, deterrol stearate, and fumitremorgin B were the metabolic biomarkers at 24 h. A total of 24 metabolites were significantly up-regulated at 48 h, in particular, nnal-N-oxide, 4-(nitrosoamino)-1-(3-pyridinyl)-1-butanone, and 9,10-epoxyoctadecanoic acid were the top 3 substrates, which had the high VIP scores. Phenylalanyl–asparagine, (E)−4–[5–(4–hydroxyphenoxy)-3-penten-1-ynyl] phenol, 9,10–epoxyoctadecanoic acid, cinnzeylanine [10]–dehydrogingerdione, (x)−2–heptanol glucoside, and blumenol C glucoside were the most abundant compounds identified at 48 h.

### Prediction of Metabolic Pathways by Identifying Significantly Different Metabolites

**Figure 4:**
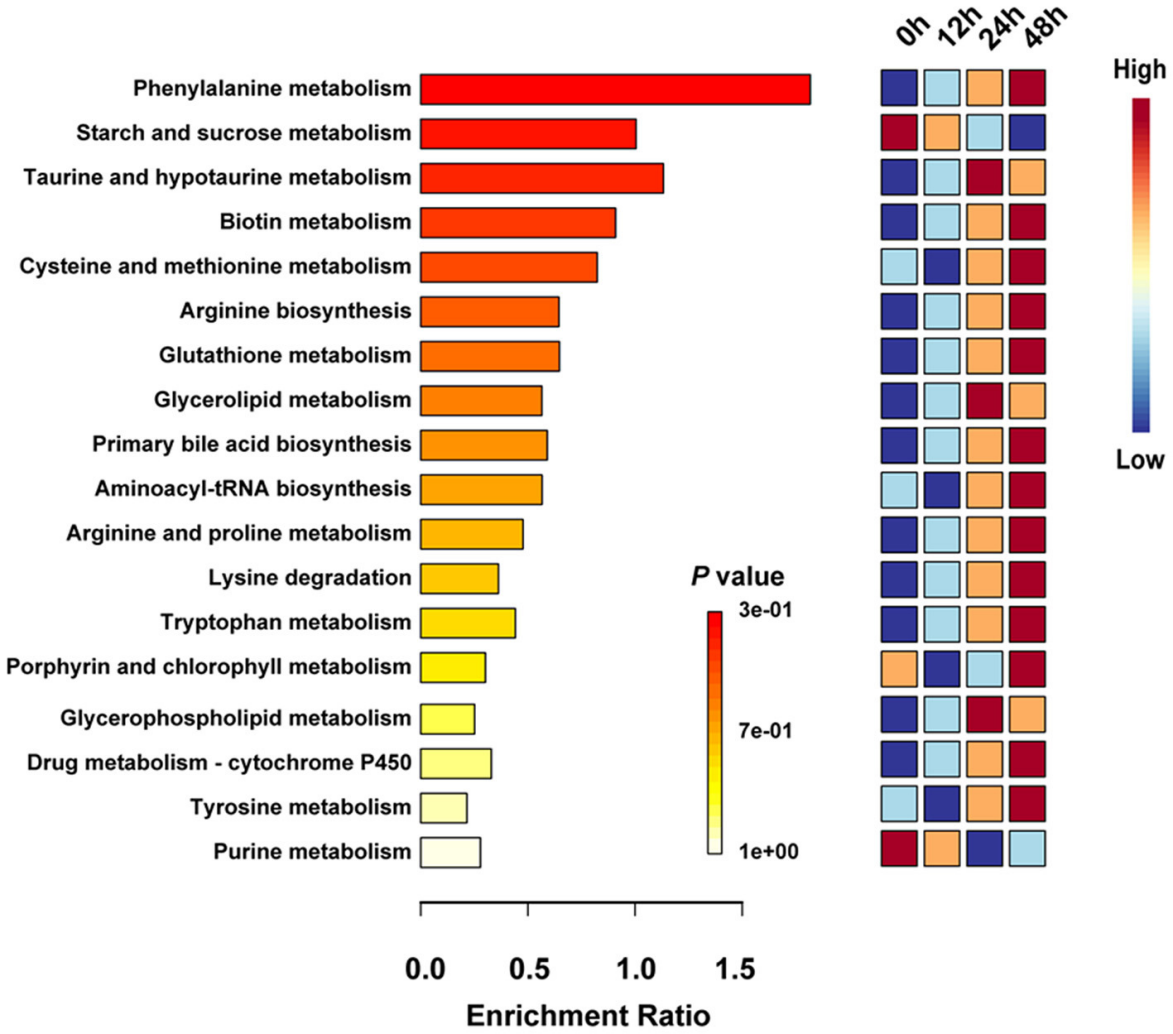
Prediction of metabolic pathways by identifying significantly different metabolites. The significantly different metabolites are based on the variable importance for the projection (VIP) >1 and *P* < 0.05.

KEGG databases were used to explore the metabolism pathway and elucidate the mechanism of metabolic changes during fermentation. Thus, enrichment analysis and topological analysis were performed to find the key pathways that were most relevant to the metabolites. A total of 22 metabolic pathways involving 229 different metabolites were sorted out throughout the fermentation process (Supplementary Tables S2, S3). To more intuitively and directly compare the differences of metabolic pathways at different fermentation time points, the first 20 metabolic pathways were retained (Figure 4). Metabolites mapped to phenylalanine metabolism, starch and sucrose metabolism, taurine and hypotaurine metabolism, biotin metabolism, and cysteine and methionine metabolism were the main metabolism pathways in the whole fermentation process. Starch and sucrose metabolism and purine metabolism enriched in 0 h and decreased until the end of fermentation. Taurine and hypotaurine metabolism, glycerolipid metabolism, and glycerophospholipid metabolism were significantly upregulated in 24 h. A total of 15 significantly different metabolic pathways were remarkably increased in 48 h, including phenylalanine metabolism, biotin metabolism, cysteine and methionine metabolism, arginine biosynthesis, and glutathione metabolism etc. These findings suggested the succession of different metabolic pathways during the process of fermentation, especially the transformation of related metabolism caused by the change of fermentation condition.

### Correlations Among the Significantly Different Microbiota, Nutritional Value, Metabolites, and Metabolic Pathways During Fermentation

**Figure 5:**
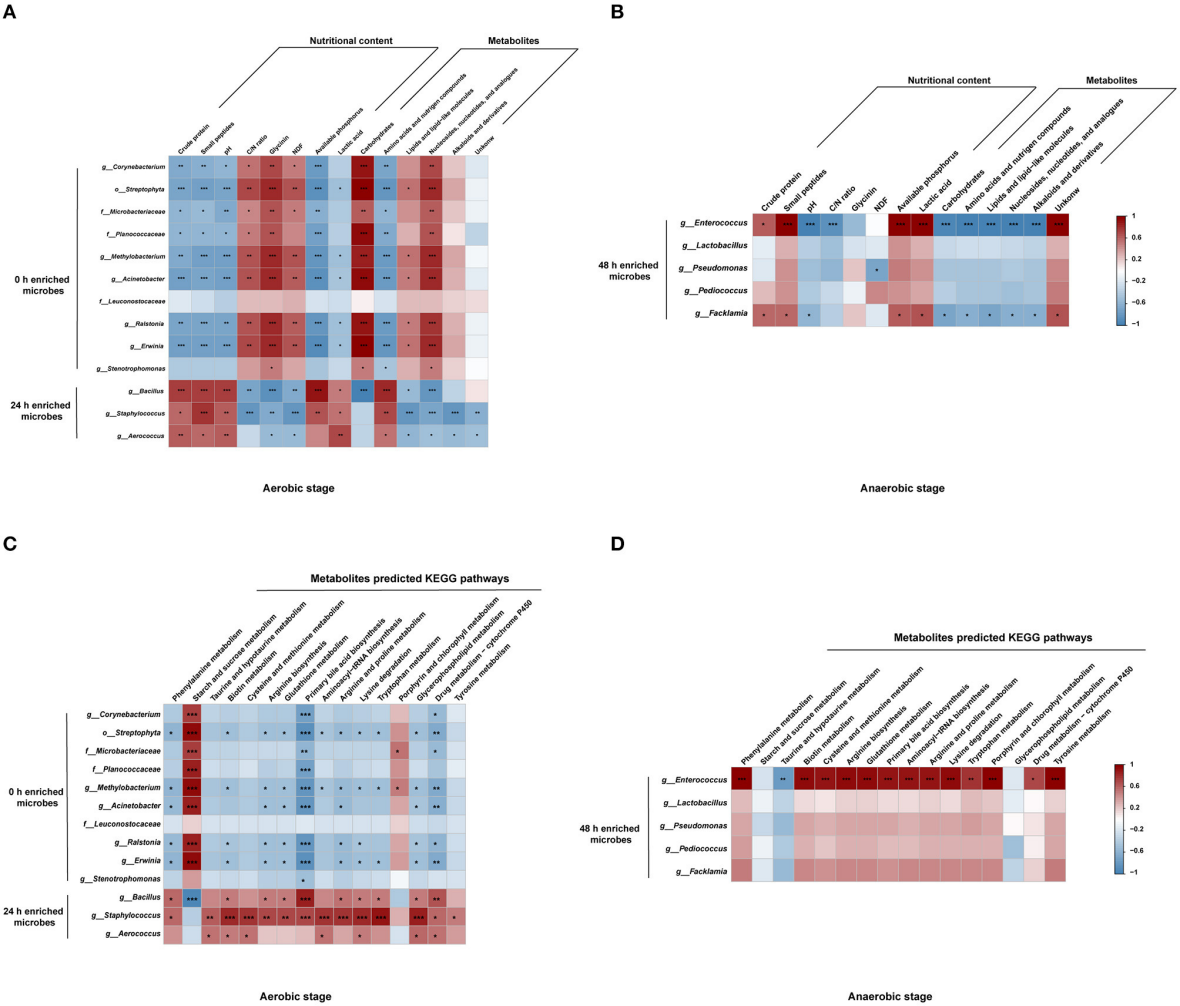
Relationships among the microbiota, metabolites, nutritional value, and KEGG pathways. Relationships among the microbiota, metabolites, and nutritional value in aerobic stage **(A)** and anaerobic stage **(B)**. Relationships between the microbiota and metabolite predicted KEGG pathways in the aerobic stage **(C)** and the anaerobic stage **(D)**. ***P < 0.001, **0.001 < *P* < 0.01, *0.01 < *P* < 0.05, respectively.

To further investigate the observations regarding the impact of the changes in the bacteria, nutritional indexes, metabolites, and metabolic pathways, correlation analyses were performed. *Bacillus, Staphylococcus*, and *Aerococcus* which increased at 24 h had the opposite relations with nutritional value and metabolites, compared with that of 0 h enriched microbes. *Bacillus, Staphylococcus*, and *Aerococcus* were positively related with amino acids and nitrogen compounds and negatively correlated with lipids and nucleosides, and contributed to the nutritional value of the substrates (Figure 5A). Notably, *Bacillus* was negatively related to carbohydrates. Correlations among the metabolites, microbiota, and nutrients in the anaerobic stage are shown in Figure 5B. *Enterococcus* and *Facklamia* had positive correlations with crude protein, small peptides, and lactic acids, and were negatively correlated with most of the metabolites. The correlations between microbes and metabolic pathways were further conducted. *Bacillus* was negatively related to starch and sucrose metabolism and positively correlated with most of the amino acid metabolism in the aerobic stage (Figure 5C). In the anaerobic stage, *Enterococcus* was positively related to amino acid metabolism (Figure 5D).

### Integrated Microbiomic and Metabolomic Changes of Functional Pathways During Fermentation

**Figure 6:**
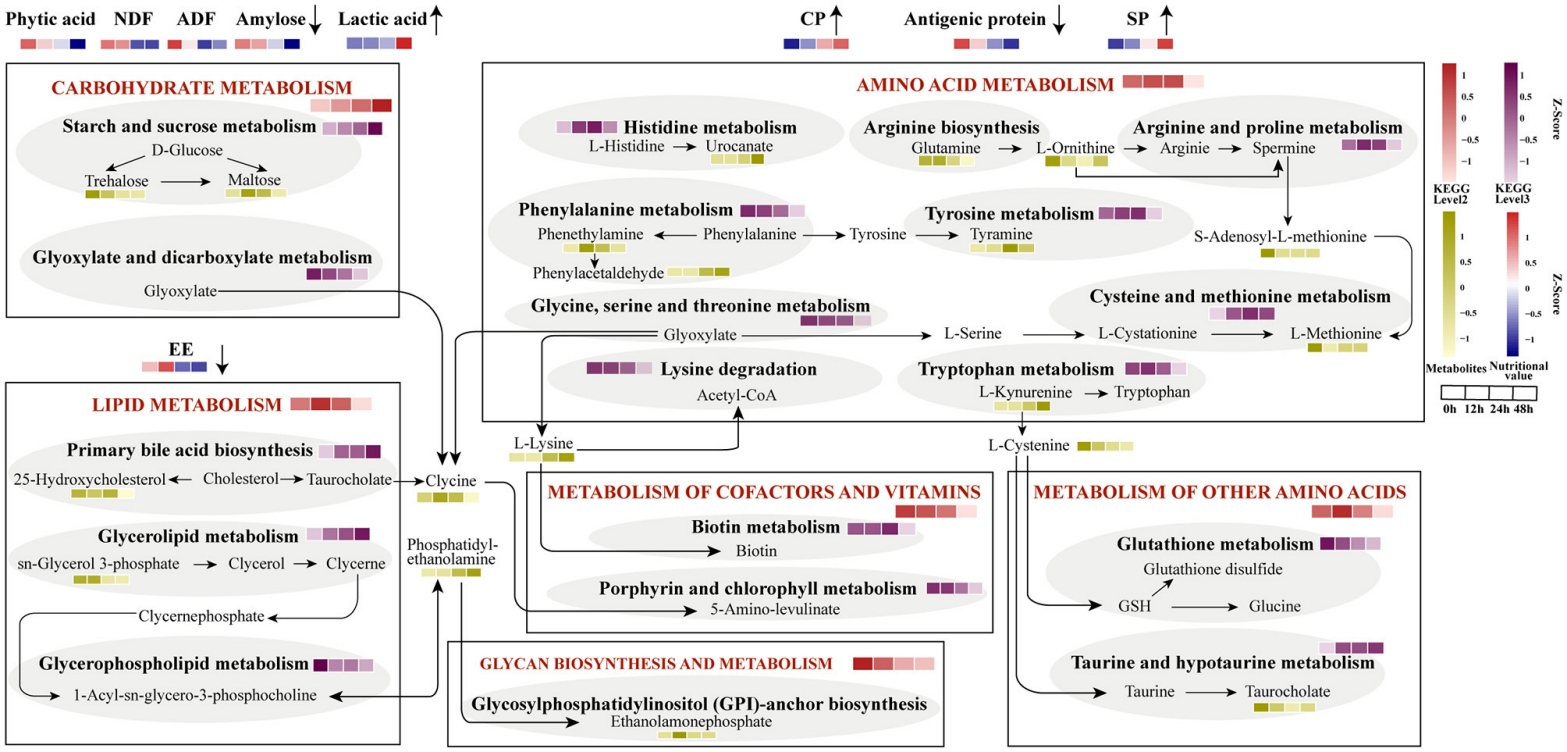
Integrated microbiomic and metabolomic changes of functional pathways. The KEGG level2 was selected based on significantly different metabolic data. The abundance of KEGG level2 and 3 were predicted by 16S data.

The integrated metabolic pathways are shown in the metabolome view map (Figure 6). A total of 6 microbial metabolism pathways based on KEGG database at level 1 were identified, including carbohydrate metabolism, lipid metabolism, amino acid metabolism, metabolism of cofactors and vitamins, metabolism of other amino acids, and glycan biosynthesis and metabolism. Carbohydrates metabolism consistently increased from the aerobic to anaerobic stages, while the metabolism of cofactors and vitamins, glycan biosynthesis, and metabolism decreased from 0 to 48 h. Lipid metabolism and metabolism of other amino acids were enriched at 12 h, amino acid metabolism increased to a high level at 24 h. At level 2, starch and sucrose metabolism and glyoxylate and dicarboxylate metabolism showed an opposite expression trend of change. Primary bile acid biosynthesis and glycerolipid metabolism consistently increased, but glycerophospholipid metabolism decreased from 0 to 48 h. C-lysine was the product of three major metabolic pathways involving carbohydrate metabolism, lipid metabolism, and amino acid metabolism, and then could be converted into 5-amino-levulinate in porphyrin and chlorophyll metabolism. Phosphatidyl-ethanolamine was produced by glycerophospholipid metabolism, and further generaterd ethanolamonephosphate which was higher at 12 h. The content of L-cysteine and taurocholate was down-regulated, and L-cysteine was the precursor of taurine which can be converted into taurocholate.

## Discussion

In recent years, fermented plant-based foods have gained attention due to their potential health benefits (28, 29). Fermented corn and soy-based foods such as fermented corn flour, Natto, Sufu, Miso, Douchi, and soy sauce are popular because they are important plant-derived food for humans and have a huge global consumption. Many research studies have reported that fermentation is capable of reducing anti-nutritional factors, making the original substrate more flavorful and nutritional. However, few studies spotlight the dynamics of fermented corn and soybean by-products by appling microbiome and matebolome. The present study indicated the temporal changes during microbial fermentation of fermented corn and defatted soybean based on using a multi-omics approach. *Bacillus, Enterococcus*, and *Pseudomonas* were mainly involved in the maturation of the fermented corn and defatted soybean. Importantly, phenylalanine metabolism was the main and vital metabolism pathway in the anaerobic fermentation stage. Correlation analysis suggested a strong relationship between *Bacillus* and amino acids in the aerobic stage, and *Enterococcus* mainly acted as an acid and amino acids producer at the anaerobic stage of fermentation.

The changes of metabolites were found to be time dependent during fermentation by adapting a two-step fermentation method. PLS-DA analysis and the structure of metabolites showed that metabolic profiles were significantly different among the groups. The microbial analysis also revealed that the dynamic changes of microbial community occurred during this two-stage solid-state fermentation process. These findings suggest that the change of metabolites during fermentation may be caused by the change of microbial niche at different stages. A recent study reported that different fermented soy foods had various soluble and volatile metabolites (30). Besides, fermenting microbes and metabolites contributes to the taste and flavor of Sufu (31). These findings have implications for considering the metabolites as a way to assess the fermented plant-based food and the metabolites can be used to understand the fermentation stages.

Metabolites annotation further indicated that metabolic activities of carbohydrates, amino acids, and lipid metabolism were dominant during fermentation, which is in agreement with previous studies about fermented soybean paste (32, 33). Amino acids, peptides, and proteins increased, whereas lipids and lipid-like molecules, and carbohydrates decreased during fermentation in our study. These results were consistent with the changes in nutrients as the content of antigenic protein, NDF, ADF, amylose, and crude fat reduced, and the level of crude protein raised during the anaerobic fermentation stage. For individual metabolites, daidzin, a natural organic compound in soybean (34), is elementary for the oxidation of acetaldehyde derived from ethanol metabolism and can be converted by resident bacteria for secondary metabolism (35). Soybean and soybean-based products are rich in daidzin, which is poorly absorbed in the human gut (36). The decrease of daidzin during the fermentation process in our study indicates that fermentation improves the bioavailability of isoflavones and assists in the digestion of protein. 5-Aminopentanoic acid is a lysine degradation product, which could be produced by bacterial catabolism of lysine (37). Nontargeted urine metabolomics analysis showed that 5-aminopentanoic acid was a biomarker, which played a role in protective and therapeutic effects on high-fat feed-induced hyperlipidemia in rats (38). The increase of 5-aminopentanoic acid suggests that the fermentation up-regulates potential benefit of the substrates. Notably, the dramatic increase in phenylacetaldehyde was observed at 48 h. Phenylacetaldehyde is a typical fragrant compound in traditional Chinese-type soy sauce and alcohol-free beers (39, 40). These results provide an evidence that important contribution of these flavor compounds to the value of fermented corn and defatted soybean.

The identification of significantly different metabolites further confirmed that fermentation caused significant changes in various metabolites. Previous studies reported that several bacterial species (*Bacillus* and *Pseudomonas)* metabolically degraded trimethylamine oxide (TMAO) in food fermentation (41). Clinical evidence supported that there is a strong association between elevated TMAO levels with increased risk of developing diseases such as atherosclerosis and thrombosis (42). The present study demonstrated that the level of TMAO continued to decrease, and *Bacillus* and *Pseudomonas* were rich in the fermentation stage. These findings are consistent with previous reports and suggested that fermentation reduces harmful substances. The source of dipeptides and free amino acids is mainly caused by protease secreted by microorganisms that decompose the protein components of a Thai fermented soybean (43). Jin et al. (44) found that L-theanine, glutamine, and glutamic acid enriched in the fermented corn, defatted soybean, and bran incubated with a combination of probiotics. Studies showed that the concentrations of most amino compounds gradually increased during fermentation with soybean as substrate (32, 45). The concentration of phenylalanyl-asparagine, protein serine, and isoleucyl-histidine showed a steady increase over time, which supports the notion of a previous study. Phenylalanyl-asparagine and isoleucyl-histidine are incomplete breakdown products of protein digestion or protein catabolism. Phenylalanine, isoleucine, and histidine are essential amino-acids. Serine, a health-promoting compound, is functionally important in many proteins which are involved in the metabolism of fats, fatty acids, cell membranes, and a healthy immune system. These metabolites outcomes directly implied the degradation of protein during fermentation and the production of amino acids, peptides, and analogs occurred and may play essential roles in quality improvement of the fermented corn and defatted soybean.

To reveal the specific effect of the significantly different metabolites during fermentation, KEGG databases were used to characterize the most influential pathways. The level of phenylalanine and glutamine was improved during the fermentation of soybean (46, 47). Tyrosine is considered to be an indispensable dietary amino acid in humans and animals, and a diet supplemented with phenylalanine is a common way of compensating it (48). Glutathione is a major antioxidant, which is metabolized in multiple ways leading to the biosynthesis of glutamate, glycine, cysteine, and other amino acids (49). The present study showed that phenylalanine metabolism, glutathione metabolism, cysteine, and methionine metabolism were the top 3 important metabolism pathways during fermentation. Our previous study also demonstrated that the content of amino acid metabolism gradually increased as fermentation progressed in terms of the predicted microbial function (24). These results indicated that the metabolism of amino acids was active and the number of amino acids increased in the fermentation of corn and defatted soybean. In addition, phenylalanine metabolism is a major pathway for anthocyanin biosynthesis (50). Anthocyanin aroused a wide public concern as a potent beneficial metabolite due to its antioxidant activity, antimicrobial, antiviral, and antithrombotic characteristics (51, 52). Anthocyanin was detected after fermentation as well, suggesting that fermentation may improve the nutritional value of the fermented substances. Therefore, these affected metabolic pathways are closely related to the fermented corn and defatted soybean and provide clues for further study on the effect of two-stage fermentation on its metabolites.

Complex microbial–ecological interactions influence the production of metabolites. In the first-stage aerobic fermentation, *Bacillus* rapidly proliferated. Relevant studies reported that *Bacillus* was the predominant fermentative bacteria responsible for the natural fermentation of protein-rich food, production of flavor substances, conversion of complex food compounds in small components in the fermentation of west African seed condiments (53). In a Nigerian fermented soybean condiment, *Bacillus* was the dominant species occurring throughout the fermentation (54). *Bacillus* was positively related to amino acids and nitrogen compounds revealing that the proteolysis of *Bacillus* results in the increase of peptides, amino acids, and ammonia from proteins. In addition, the degradation of complex carbohydrates from the enzymes secreted by *Bacillus*, such as amylase, galactanase, galactosidase, glucosidase, and fructofuranosidase, interpreted the positive correlation between *Bacillus* and carbohydrates in the aerobic stage. *Enterococcus*, a lactate-producing bacteria as well as an acid-tolerant bacteria (55, 56), plays important roles in food production, particularly they can accelerate the ripening of food and improve the taste and flavor through proteolysis, lipolysis, carbohydrates breakdown, and the production of aromatic compounds (57). *Enterococcus* was the most dominant genus in the anaerobic fermentation stage. The enzyme secreted by *Enterococcus* and *Bacillus* played important roles in hydrolyzing proteins, and the accumulation of amino acids and related substances occurred in the whole process, resulting in a positive correlation between *Enterococcus* and amino acids. Small peptides in foods are generally easy to be absorbed and utilized by the gut, and most of them have specific biological functions (58–60), such as improving gut health and immunity. The relationships of microbes and metabolic pathways further confirmed the effects on protein and carbohydrates breakdown of *Bacillus* and amino acid synthesis of *Enterococcus*. A detailed understanding of the fermentation microbiota and their unique functional characteristics is fundamental to developing high-quality and safe fermented products and enhancing specifically adapted starter cultures. These results implied that *Bacillus* and *Enterococcus* were critical bacteria in corn and defatted soybean fermentation and suggested that they may be the optimized selective strains used in the two-stage fermentation.

The overview of the metabolic pathways map will help to further explain the causes of the differences in these metabolites and to explore the metabolic mechanism. Interestingly, the content of amino acid metabolism increased at the aerobic phase but declined abruptly at 48 h, and the consistent increase of carbohydrates was observed according to the predicted microbial functions. These findings were distinct from the metabolomics analysis, suggesting the limitation and deviation of the analysis of the dynamics during the fermentation based solely on microbial data. In the first stage, bacteria like *Bacillus* which directly participated in amino acid metabolism resulted in increased metabolic capacity. With the proliferation of carbohydrate-degrading bacteria and the increased acidification in the second stage, the main metabolism of fermentation gradually changes to carbohydrate metabolism. Therefore, the distinctive dominant metabolic functions at different fermentation stages were further interpreted. The physicochemical features were consistent with the change of metabolites, verified the metabolism differences caused by the significantly different bacteria in the two-stage fermentation. The metabolic process of fermented plant-based food is complex, and the specific metabolic mechanisms of these metabolites identified in our study are still not fully understood. Thus, future studies should focus on the secondary metabolites and establish standardized metabolic fingerprints for the fermentation.

## Conclusions

In summary, the dynamic changes in the nutritional properties, microbial composition, and metabolites during the corn and defatted soybean fermentation were systematically studied. Phenylalanine metabolism, glutathione metabolism, and cysteine and methionine metabolism were considered to be the important metabolic pathways affecting the quality of fermented corn and defatted soybean. The results further unraveled that *Bacillus* spp. was the predominant genera that mainly participated in the breakdown of protein and carbohydrates in the aerobic stage, and *Enterococcus* spp. was associated with amino acid metabolism and lactic acid production toward the end of fermentation. This study potentially serves as a foundation for increasing the nutrition of corn and defatted soybean food and guides the underlying fermenting mechanism of solid-state fermentation of plant-based food.

## Data Availability Statement

The datasets presented in this study can be found in online repositories. The names of the repository/repositories and accession number(s) can be found in the article/Supplementary Material.

## Author Contributions

CW and YW conceived the project and designed the experiments. CW and SW analyzed the nutritional value, 16S rRNA sequencing data and metabolic data, and wrote the manuscript. MJ, MY, and YW help review, revise, and approve the manuscript. BL assisted the nutritional value analyses and contributed to revise and approve the manuscript. All authors contributed to the article and approved the submitted version.

## Funding

This work was funded by China Agriculture Research System of MOF and MARA (CARS-35).

## Conflict of Interest

The authors declare that the research was conducted in the absence of any commercial or financial relationships that could be construed as a potential conflict of interest.

## Publisher's Note

All claims expressed in this article are solely those of the authors and do not necessarily represent those of their affiliated organizations, or those of the publisher, the editors and the reviewers. Any product that may be evaluated in this article, or claim that may be made by its manufacturer, is not guaranteed or endorsed by the publisher.

## Supplementary Material

Supplementary Figure S1 Dimension reduction analysis and clustering of dynamic metabolome. **(A)** Principal component analysis (PCA) plot of compounds in fermented mixed substances. **(B)** Partial Least Squares Discriminant Analysis (PLS-DA) plot of compounds in fermented mixed substances. The accuracy, goodness-of-fit (R2), and goodness-of-prediction (Q2) were 1.0, 0.999, and 0.991, respectively. **(C)** Orthogonal partial least-squares discrimination analysis (OPLS-DA) plot of compounds in fermented mixed substances; R2X, R2Y, and Q2 were 0.098, 0.883, and 0.523, respectively. **(D)** Cluster analysis of different fermentation times.

Supplementary Table S1 Determined nutrient contents of fermented corn and defatted soybean at 0, 12, 24, and 48 h.

Supplementary Table S2 Identification of significantly different metabolites during fermentation.

Supplementary Table S3 Enrichment pathways of significantly different metabolites in fermented mixed substances.
