# Supplementary figures and images for: Integrated Microbiomic and Metabolomic Dynamics of Fermented Corn and Soybean By-Product Mixed Substrate
Source: Front Nutr. 2022 Feb 28;9:831243. doi: 10.3389/fnut.2022.831243 (PMC8922052; doi:10.3389/fnut.2022.831243)

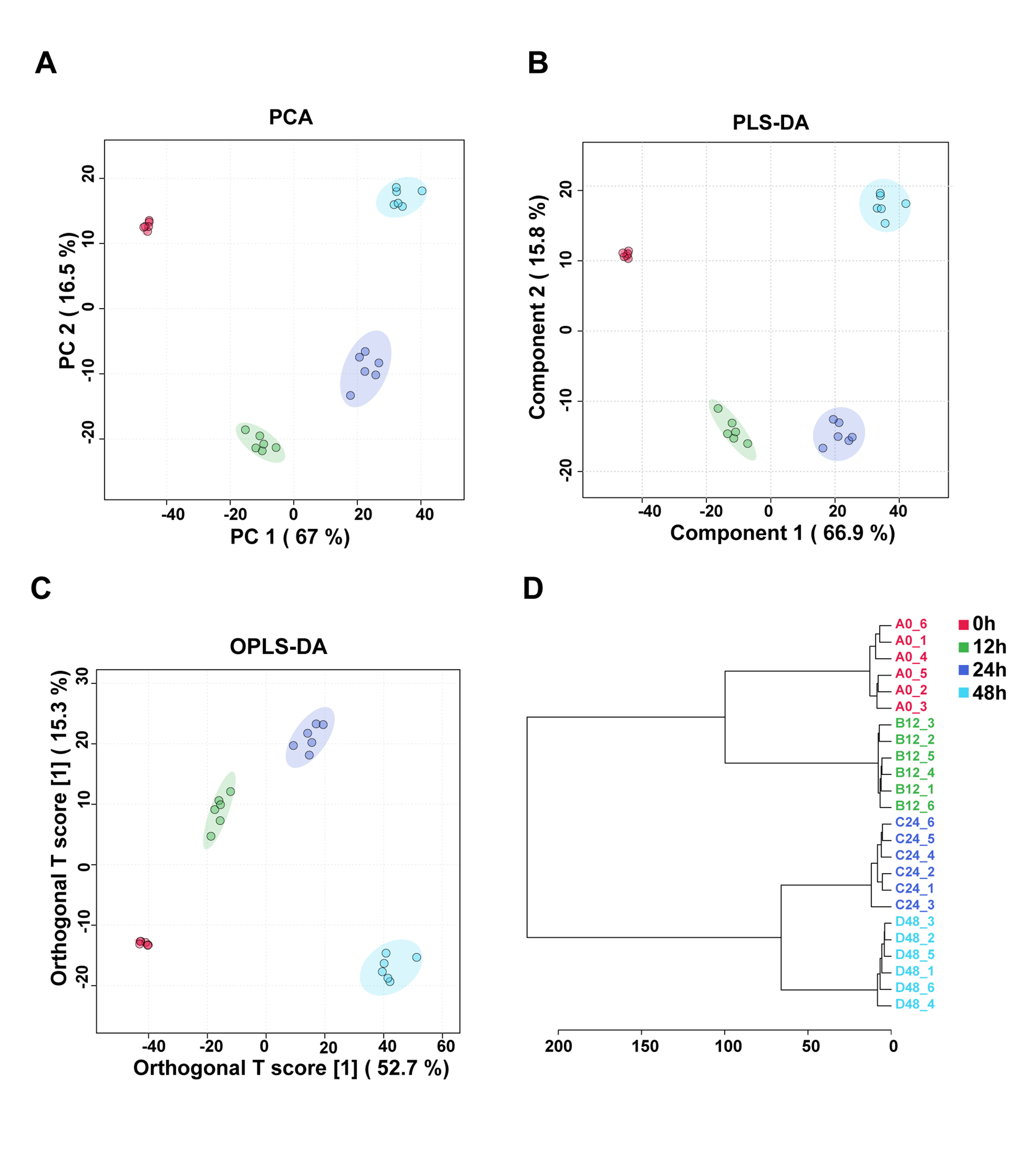

Supplement: Supplementary Figure S1 — Dimension reduction analysis and clustering of dynamic metabolome. (A) Principal component analysis (PCA) plot of compounds in fermented mixed substances. (B) Partial Least Squares Discriminant Analysis (PLS-DA) plot of compounds in fermented mixed substances. The accuracy, goodness-of-fit (R2), and goodness-of-prediction (Q2) were 1.0, 0.999, and 0.991, respectively. (C) Orthogonal partial least-squares discrimination analysis (OPLS-DA) plot of compounds in fermented mixed substances; R2X, R2Y, and Q2 were 0.098, 0.883, and 0.523, respectively. (D) Cluster analysis of different fermentation times. [file Image_1.TIF]
